# The Impact of Adherence to Screening Guidelines and of Diabetes Clinics Referral on Morbidity and Mortality in Diabetes

**DOI:** 10.1371/journal.pone.0033839

**Published:** 2012-04-03

**Authors:** Carlo Giorda, Roberta Picariello, Elisa Nada, Barbara Tartaglino, Lisa Marafetti, Giuseppe Costa, Roberto Gnavi

**Affiliations:** 1 Metabolism and Diabetes Unit, ASL TO5, Regione Piemonte, Chieri, Italy; 2 Epidemiology Unit, ASL TO3, Regione Piemonte, Grugliasco, Italy; 3 Chaira Medica Association, Chieri, Italy; 4 Department of Public Health, University of Torino, Torino, Italy; Postgraduate Medical Institute & Hull York Medical School, University of Hull, United Kingdom

## Abstract

Despite the heightened awareness of diabetes as a major health problem, evidence on the impact of assistance and organizational factors, as well as of adherence to recommended care guidelines, on morbidity and mortality in diabetes is scanty. We identified diabetic residents in Torino, Italy, as of 1st January 2002, using multiple independent data sources. We collected data on several laboratory tests and specialist medical examinations to compare primary versus specialty care management of diabetes and the fulfillment of a quality-of-care indicator based on existing screening guidelines (GCI). Then, we performed regression analyses to identify associations of these factors with mortality and cardiovascular morbidity over a 4 year- follow-up. Patients with the lowest degree of quality of care (i.e. only cared for by primary care and with no fulfillment of GCI) had worse RRs for all-cause (1.72 [95% CI 1.57–1.89]), cardiovascular (1.74 [95% CI 1.50–2.01]) and cancer (1.35 [95% CI 1.14–1.61]) mortality, compared with those with the highest quality of care. They also showed increased RRs for incidence of major cardiovascular events up to 2.03 (95% CI 1.26–3.28) for lower extremity amputations. Receiving specialist care itself increased survival, but was far more effective when combined with the fulfillment of GCI. Throughout the whole set of analysis, implementation of guidelines emerged as a strong modifier of prognosis. We conclude that management of diabetic patients with a pathway based on both primary and specialist care is associated with a favorable impact on all-cause mortality and CV incidence, provided that guidelines are implemented.

## Introduction

Because of its toll in terms of morbidity and mortality for millions of people all over the world, diabetes is a major concern for National Health Systems [1], [2]. Recall of patients and processes of screening such as hemoglobin A1c and lipid determination, blood pressure measurement and annual eye and albuminuria screening have proved to be effective in identifying and treating patients at risk [3], [4]. However, worldwide, the quality of care of persons with diabetes, and intermediate or long-term outcomes of the disease, are rather unsatisfactory and variable [5], [6]. These differences are influenced by a complex web of factors, in which health care organization plays an important role. Unstructured care in the community is associated with poorer follow up, greater mortality and worse glycaemic control than hospital care. [7]. Organizational factors in diabetes care, in the long and medium term, can greatly affect the prognosis of patients as regards survival [8], morbidity and hospital utilization [9].

A surveillance population-based programme monitoring diabetes through the employment of multiple data sources has been implemented in the city of Torino, in north-western Italy. Recently the programme has allowed to estimate the quality of care process in terms of adherence to recommended guidelines (GL) for monitoring of diabetes. On these premises the latter survey revealed greater adherence to guidelines in patients cared for by both specialist and primary care compared to those only seen by General Practitioner (GP) [10].

Consequent to these previous findings, we investigated the hypothesis that these differences in the type of care and adherence to screening guidelines might have any impact on several hard outcomes such as mortality and incidence of major cardiovascular events.

## Methods

### Study population

**Figure 1:**
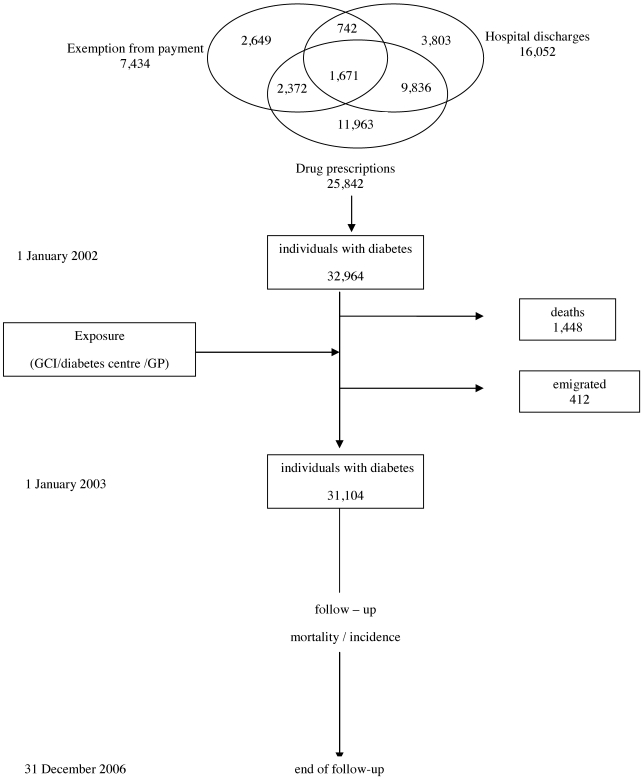
Source of ascertainment of people with diabetes and time windows used for exposure assessment and for follow-up.

The study base included residents in the city of Torino (900,000 inhabitants) at 1 January 2003, aged = 20 years, with a diagnosis of diabetes. No ethical approval was requested according to Italian law 211/2003 which explains that no ethic committee's permission is required for this kind of studies in Italy (anonymous aggregated data). As described in detail elsewhere [11], [12], patients were identified using three data sources: the first source was the file of all residents discharged from hospitals with a primary or secondary diagnosis of diabetes from January 1, 1997 to December 31, 2001. The second data source was the file of prescriptions for anti-diabetic drugs prescribed to residents from January 1 to December 31, 2001; we considered as persons with diabetes only those who had at least two prescriptions of anti-diabetic drugs. The third source was the file of all subjects who obtained exemption from payment of drugs, syringes, and glucose monitoring strips due to a diagnosis of diabetes from January 1, 1998 to December 31, 2001. All data sources were matched by a deterministic linkage procedure using a unique identifier; the study population included all persons who were present in at least one of three health data sources (Figure 1). This database was further linked to the Torino Population Register to include only people alive on 1 January, 2002 and to determine each individual's educational level. Treatment was classified into three groups: diet only, oral antidiabetic drugs, and insulin. Information about therapy was retrieved either from the RDR, or from prescriptions of antidiabetic drugs. Subjects who were prescribed both insulin and oral antidiabetic drugs were assigned to “insulin treatment”; all diabetic people who were not registered in the RDR and had not received any antidiabetic drug prescription were considered within the “diet only treatment” group. We considered all those discharged from a hospital in the previous five years with a diagnosis of coronary heart disease, cerebrovascular disease or disease of arterie as individuals with established cardiovascular disease (CVD).

### The levels of care

All Italian citizens are cared for by a GP as part of the National Health System (NHS). Specialist care for individuals with diabetes is provided mainly by a public network of 700 diabetes clinics (14 in Torino), delivering diagnostic confirmation, therapy, counseling on healthy life styles, and early diagnosis of complications, through close patient follow-up by a diabetes team of professionals, including diabetologists, and the scheduling of regular check-ups. Most patients are referred to these care units by their GP and care is free. All laboratory tests and specialist medical examinations reimbursed by the NHS from 1 January 2002 to 31 december 2002 were linked to the population with diabetes (Figure 1). Accordingly, we were able to identify the Guidelines Composite Indicator (GCI), a measure which includes annual assessment of A1C and at least two assessments from among eye examinations, total serum cholesterol, and microalbuminuria. GCI can be considered a proxy of fair adherence to screening guidelines (9). We considered all individuals who had at least one consultation by a diabetologist from 1 January 2002 to 31 december 2002 as cared for by a diabetes center, whereas those who had not were considered as cared for by a GP only, as 99.4% had at least one contact with a GP in the same period. The study population was classified according to four “levels of care”: the “ONLY GP” level (patients seen by GP but not at diabetes clinics and with no fulfillment of GCI, i.e. poor adherence to GL), the “GP AND SPECIALIST, WITHOUT GCI” level (patients seen by GP and at diabetes clinics but with no fulfillment of GCI, i.e. poor adherence to GL), the “GP AND GCI, WITHOUT SPECIALIST” level (patients seen by GP and not at diabetes clinics but with fulfillment of GCI, i.e. fair adherence to GL) and the “GP AND SPECIALIST, WITH GCI” pathway (patients seen by GP and at diabetes clinics and with fulfillment of GCI, i.e. fair adherence to GL). We chose, as the reference group, the one with the highest level of care, that is diabetes clinic plus adherence to guidelines, because it is the standard care in the Region. Furthermore, it is the same mode of analysis of a similar previous paper on quality of process care [10].

### Outcomes

Subjects were followed up for mortality, incidence of acute myocardial infarction (AMI) and stroke, and non-traumatic lower extremity amputations (LEA). Information on causes of death, from the local mortality registries, was classified according to the International Classification of Diseases, 9th Revision (ICD-9): cardiovascular disease (390–459), coronary heart disease (CHD; 410–414), cerebrovascular disease (stroke; 430–438), and cancer (140–208).

AMI incident cases were identified through hospital and causes-of death-registries [13]. Hospital discharges with ICD9-CM code 410* as primary discharge diagnosis, or as secondary diagnosis when associated with selected codes suggestive of ischemic symptoms in primary diagnosis, and deaths with the ICD9 code 410* as underlying cause were selected. Individuals without a previous hospitalization for ICD9-CM codes 410* or 412* during the previous 60 months were considered as incident cases [12], [14].

Acute stroke incident cases were identified through hospital and causes-of-death registries [13]. Hospital discharges with ICD9-CM codes 430*, 431*, 434*, and 436* as primary discharge diagnosis, excluding patients with 438* code in secondary diagnosis, and deaths with ICD9 codes 430*, 431*, 434*, and 436* as underlying cause were selected. Individuals without a previous hospitalization for stroke diagnosis during the previous 60 months were considered as incident cases [12], [14].

LEA were identified using hospital discharges records with a DRG code of 113, 114, or 285. We considered the first discharge for each subject in the period 2003–2006.

### Statistical analysis

The start of follow-up was defined as January 1, 2003, excluding all persons who died or moved out of Turin in 2002, and ended at the date of incidence, death, transfer out of the area of residence, or December, 31, 2006. We considered as lost to follow-up people who moved out of Turin during the study period (4.3%) (Figure 1).

Days of follow-up were calculated as the difference between January 1, 2003, and the date of the event under study, loss to follow-up, death (if incidence was under study) or December 31, 2006, where appropriate. Person-time was calculated separately for each event; for example, when AMI incidence was studied, days of follow up were calculated until the occurrence of first AMI, and, in case a stroke occurred before AMI, the former was not considered (i.e. observation was not censored because of the stroke). Mortality/incident rates were calculated by dividing the number of death/incident cases by the total person-time and expressed in terms of event per 1,000 person-years. Both mortality and incidence density were standardized on the age distribution of the 2008 local population (5-years age classes, from 20–24 to >84). Cumulative survival probabilities according to pathways of care were estimated with the Kaplan-Meier method and compared using the log-rank test. Poisson regression was used to estimate, by each levels of care, adjusted rate ratios (RR) for available potential predictors of death or incidence: age (21–44; 45–54; 55–64; 65–74; 75–84; >84 years), gender, educational level, treatment (insulin, oral antidiabetic treatment, diet only), previous history of CVD, and Local Health Unit of residence (4 in Turin). The statistical analyses were conducted using the SAS System, version 9.1.

## Results

We identified 31104 persons with diabetes (≥20 years) resident in Torino on 1 January 2003, whose diagnosis of diabetes was already confirmed on 1 January 2002. Baseline characteristics of the study population according to the levels of care are shown in table 1. Less than 20% of patients were seen both by a diabetes clinic and received a screening for complications, while more than one third of patients diabetes clinic's consultation did not result in a basic screening for complications (GP AND SPECIALIST, WITHOUT GCI group). About 40% of patients were not cared appropriately, as they were neither seen by a diabetes clinic, nor appropriately screened for complications, while 6% were appropriately cared by a GP without consultation from a diabetologist. Persons cared with poor adherence to GL (ONLY GP group) were more likely to be old, with no pharmacological treatment and with cardiovascular disease, while patients belonging to the three other groups showed only slight socio-demographic and clinical differences.

**Table 1:** Characteristics of the study population Torino, 1 January 2003.

		Level of care				
		A	B	C	D	Total
Characteristic		Number (%)				
**All**		6084	10997	1950	12073	31104
**Gender**	Women	2957 *(48.6)*	5568 (*50.6)*	917 *(47.0)*	6133 *(50.8)*	15575 *(50.1)*
	Men	3127 *(51.4)*	5429 *(49.4)*	1033 *(53.0)*	5940 *(49.2)*	15529 *(49.3)*
**Age**	21–44	215 *(3.5)*	385 *(3.5)*	111 *(5.7)*	744 *(6.2)*	1455 *(4.7)*
	45–54	576 *(9.5)*	869 *(7.9)*	212 *(10.9)*	1088 *(9.0)*	2745 *(8.8)*
	55–64	1785 *(29.3)*	2450 *(22.3)*	533 *(27.3)*	2433 *(20.2)*	7201 *(23.2)*
	65–74	2428 *(39.9)*	3999 *(36.4)*	697 *(35.7)*	3519 *(29.2)*	10643 *(34.2)*
	75–84	988 *(16.2)*	2739 *(24.9)*	365 *(18.7)*	3078 *(25.5)*	7170 *(23.1)*
	> = 85	92 (1.5)	555 (5.05)	32 (1.6)	1211 (10.0)	1890 (6.1)
**Educational level**	High	669 (*11.0)*	1388 *(12.6)*	367 *(18.8)*	2138 *(17.7)*	4562 *(14.7)*
	Average	1769 *(29.1)*	3044 *(27.7)*	624 *(32.0)*	3481 *(28.8)*	8918 *(28.7)*
	Low	3646 *(59.9)*	6565 *(59.7)*	959 *(49.2)*	6454 *(53.5)*	17624 *(56.7)*
**Treatment**	Diet	713 *(11.7)*	1318 *(12.0)*	246 *(12.6)*	3017 *(25.0)*	5294 *(17.0)*
	Oral drugs	3656 *(60.1)*	6530 *(59.4)*	1205 *(61.8)*	7093 *(58.8)*	18484 *(59.4)*
	Insulin	1715 *(28.2)*	3149 *(28.6)*	499 *(25.6)*	1963 *(16.3)*	7326 *(23.6)*
**Cardiovascular disease**	Yes	942 *(15.5)*	2036 *(18.5)*	308 *(15.8)*	2526 *(20.9)*	5812 *(18.7)*
	No	5142 *(84.5)*	8961 *(81.5)*	1642 *(84.2)*	9547 *(79.1)*	25292 *(81.3)*
**GCI components**	A1C	6084 *(100)*	8226 *(74.8)*	1950 *(100)*	2912 *(24.1)*	19172 *(61.6)*
	Total serum cholesterol	6026 *(99.1)*	6095 *(55.4)*	1909 *(97.9)*	3911 *(32.4)*	17941 *(57.7)*
	Microalbuminuria	5091 *(83.7)*	437 *(4.0)*	1724 *(88.4)*	291 *(2.4)*	7543 *(24.3)*
	Eye examination	2559 *(42.1)*	1906 *(17.3)*	810 *(41.5)*	808 *(6.7)*	6083 *(19.6)*

A = GP and Specialist, with GCI; B = GP and Specialist, without GCI; C = GP and GCI, without Specialist; D = Only GP.

**Figure 2:**
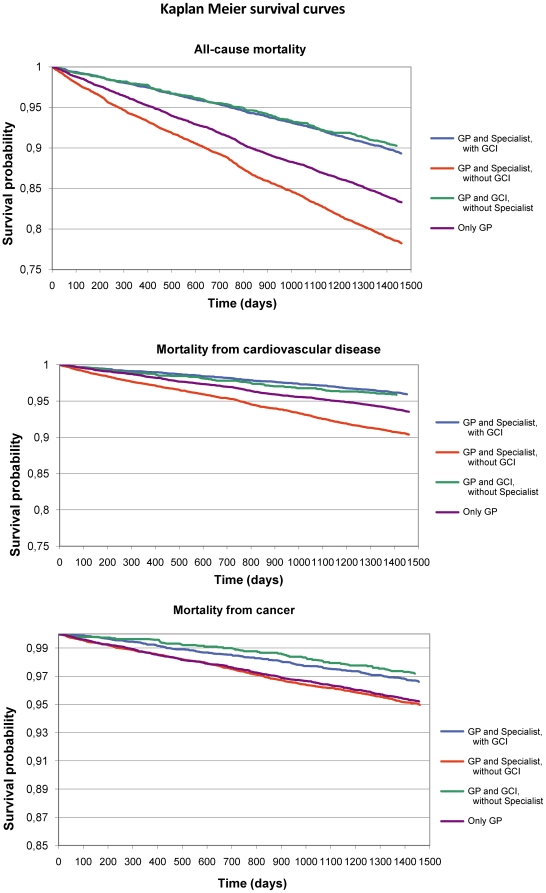
Kaplan Meier survival curves of different mortality causes.

During 4-year follow-up all cause mortality was 46.7 per 1,000 person-years (18.2 from cardiovascular disease), AMI, stroke and LEA incidence were, respectively, 10.8, 9.4 and 1.5 per 1,000 person-years. Both all cause and cardiovascular age-adjusted mortality were higher in the ONLY GP group compared to other pathway of care, but differences were less evident when compared to the GP AND SPECIALIST, WITHOUT GCI group. A slight difference in this pattern was found in cancer mortality with the GP, WITHOUT SPECIALIST AND GCI group characterized by the lowest rate. As for incidence, differences between levels of care were less marked, even if the GP AND SPECIALIST, WITH GCI group almost constantly showed the lowest risks (table 2). Unadjusted Kaplan-Meier curves showed that the GP AND SPECIALIST, WITH GCI (and GP, WITHOUT SPECILALIST AND GCI) level was associated with a significant (p<0.0001) lower likelihood of mortality, both from all-causes and from cardiovascular disease and cancer (figure 2).

**Table 2:** Number, age-standardized mortality and incidence rate (×1.000 person/year) and 95% confidence intervals for outcomes by level of care; 2003–2006.

		Level of care										
		A	B	C	D	Total						
		*n*	*std rate (95%CI)*	*n*	*std rate (95%CI)*	*n*	*std rate (95%CI)*	*n*	*std rate (95%CI)*	*n*	*crude rate (95%CI)*	*std rate (95%CI)*
**Mortality**	All causes	638	19.1 (17.2–21.3)	1798	26.0 (24.4–27.7)	185	19.9 (16.2–24.4)	2559	31.3 (29.8–32.8)	5180	46.7 (45.4–47.9)	27.0 (26.1–28.0)
	Cardiovasc. disease	235	7.3 (6.2–8.5)	657	9.4 (8.6–10.3)	77	7.4 (5.5–10.0)	1055	12.4 (11.6–13.4)	2024	18.2 (17.5–19.0)	10.5 (10.0–11.1)
	CHD	103	3.0 (2.4–3.8)	267	3.8 (3.3–4.4)	42	3.6 (2.5–5.2)	349	4.2 (3.7–4.8)	761	6.9 (6.4–7.4)	3.9 (3.6–4.2)
	Stroke	58	1.8 (1.3–2.5)	152	2.2 (1.8–2.7)	14	2.0 (1.0–4.1)	339	3.8 (3.4–4.3)	563	5.1 (4.7–5.5)	2.9 (2.7–3.2)
	Cancer	195	5.3 (4.4–6.4)	484	6.5 (5.7–7.4)	51	4.8 (3.4–7.0)	538	6.6 (6.0–7.2)	1268	11.4 (10.8–12.1)	6.2 (5.8–6.6)
**Incidence**	AMI	192	6.2 (5.0–7.6)	451	7.1 (6.1–8.2)	70	7.6 (5.4–10.8)	467	7.5 (6.5–8.5)	1180	10.8 (10.2–11.4)	7.0 (6.4–7.6)
	Stroke	163	4.6 (3.7–5.7)	381	5.9 (5.0–7.0)	39	4.1 (2.5–6.5)	452	5.7 (5.1–6.4)	1035	9.4 (8.9–10.0)	5.4 (5.0–5.9)
	LEA	24	0.5 (0.3–0.9)	70	1.3 (0.7–2.2)	8	1.7 (0.5–5.6)	67	1.0 (0.7–1.4)	169	1.5 (1.3–1.8)	1.1 (0.8–1.4)

A = GP and Specialist, with GCI; B = GP and Specialist, without GCI; C = GP and GCI, without Specialist; D = Only GP.

Table 3 shows the RRs for the outcomes considered, adjusted for age, gender, educational level, Local Health Unit of residence, cardiovascular disease and treatment. All of them showed a very similar pattern: worse outcomes in the ONLY GP group, intermediate in the GP AND GCI, WITHOUT SPECIALIST group whereas the best outcomes could be found in the GP AND SPECIALIST, WITH GCI group. Interestingly, diabetologist's consultation added little to adherence to guidelines, since the RR of the small GP AND GCI, WITHOUT SPECIALIST group is not significantly different from that of the GP AND SPECIALIS, WITH GCI group. With respect of mortality, a consistent added value of diabetologist consultation, independent of guidelines implementation, stood out in the comparison between the GP AND SPECIALIST, WITHOUT GCI and the ONLY GP groups.

**Table 3:** Rates ratios (RR) and 95% confidence intervals for mortality and for incidence of major cardiovascular events by level of care; 2003–2006.

		Level of care			
		Specialist and GP, with GCI	Specialist and GP, without GCI	GP and GCI, without Specialist	Only GP
		RR	RR (95% CI)	RR (95% CI)	RR (95% CI)
**Mortality**	All causes	1	1.29 (1.17–1.41)	0.95 (0.81–1.12)	1.72 (1.57–1.89)
	Cardiovascular disease	1	1.19 (1.03–1.38)	1.06 (0.82–1.37)	1.74 (1.50–.2.01)
	CHD	1	1.16 (0.93–1.46)	1.31 (0.91–1.88)	1.48 (1.18–1.86)
	Stroke		1.04 (0.76–1.40)	0.77 (0.43–1.38)	1.93 (1.44–2.57)
	Cancer	1	1.26 (1.07–1.50)	0.86 (0.63–1.17)	1.35 (1.14–1.61)
**Incidence**	AMI	1	1.24 (1.04–1.47)	1.22 (0.92–1.60)	1.31 (1.10–1.55)
	Stroke	1	1.14 (0.95–1.38)	0.77 (0.54–1.09)	1.32 (1.09–1.59)
	LEA	1	1.57 (0.99–2.50)	1.15 (0.51–2.56)	2.03 (1.26–3.28)

## Discussion

The main finding of our study is the link between some assistance and organizational factors (type and quality of assistance) and hard outcomes of diabetes. Compared with those followed with the highest quality of care, patients who had been managed in an old-fashion, unstructured way (i.e. no planned screening and no diabetes clinic referrals), had excess all-cause mortality (RR 1.72), and excess incidence of cardiovascular events (RRs for AMI 1.31, for stroke 1.32 and for LEA 2.03). These trends are consistent throughout the outcomes considered: mortality appeared to be increased not only for cardiovascular diseases, but also for cancer; incidence of major cardiovascular events, consistently increased for myocardial infarction, stroke and amputation, mirrors the pattern of mortality, even if at a lower scale. While the relation between diabetes and cardiovascular disease has been well known for long time, nowadays also the link between diabetes and cancer is established to such an extent, that cancer can be viewed as a new chapter in the field of diabetes complications [15]. Also in this population the risk of death from cancer for people with diabetes, compared with those without diabetes, was increased of 40% in both genders [13]. Moreover, there appear to be a protective effect of aggressive diabetes treatment on cancer development: previous observations in the Verona Study [8] had suggested lower cancer mortality in diabetics on diet seen at diabetes clinics (Verlato, personal communication) and metformin has been shown to have a protective effect on tumors occurrence [16]. In our study the protective effect of diabetes clinic consultation is confirmed in addition to a novel piece of news, i.e. this result is even more apparent when there is a good implementation of GL. Besides metformin use, lifestyle modifications, that are usually suggested and enforced in more structured models of care as a tool for better metabolic control and CV prevention, and/or the routine periodical encounter and interview with a doctor, may be responsible for this finding.

Consistently with other studies, diabetes clinic referral [8], [11], [17], [18] emerges as a good predictor of better long-term prognosis, being itself associated with a reduction of the probability of death by more than 33%. Compared with those who are only cared for by other physicians, patients seen at a diabetes center are more likely to be monitored according to guidelines, regardless of severity-of-disease effect [10], and to receive structured education as well as more aggressive treatment when needed [19]. In our results this property is still retained by diabetologists, but it is dampened when the consultation does not result in a sufficient adherence to guidelines. From the point of view of the care system this is valuable, new information.

The impact of GLs implementation in diabetes management on hard outcomes such as death and incidence of chronic complications is still controversial. De Belvis et al. have found that adherence to EBM instruments is likely to improve process of care, rather than patient outcomes [20]. Other authors [21] have evaluated the effect of GLs on intermediate or process indicators concluding that a quality improvement program improved the provision of diabetes care but was not accompanied by any effect on patient outcomes. When it comes to hard end points, such as mortality or incidence of CVD, particularly for short periods of time, the available evidence becomes weak. Recently, several investigations have explored the impact of GLs on morbidity: a favorable impact of GL on development of macrovascular complications [22] and a positive relationship between good performance of doctors in process indicators and reduction of cardiovascular events over time have been described [23]. Both suggest some evidence in favor of adherence to guidelines, but these surveys refer either to patients cared for by diabetes clinics or to selected cohorts, and not to the general population of individuals with diabetes. A strength of our study is that, through record linkage between several data sources, we were able to accurately monitor the care process and outcomes longitudinally at the population level. Throughout all the analysis of the Torino Study, adherence to GLs emerged as a strong modifier of prognosis in the diabetic population of the city, therefore, to the best of our knowledge, this is the first confirmation that the link between diabetes clinic referral and adherence to GLs in a large unselected population with diabetes has favorable impact on mortality. Finally, we hold that the findings of this study can be generalised to other health system as the protective effect over adverse health outcomes of adherence to guide lines is not dependent on the heath system. Moreover, diabetologists and diabetes clinics exist in all developed nations. Referring patients to diabetes clinics for diabetes may be easier in some countries but it is possible in most of health systems.

Our study has limitations that could affect the results. Available administrative databases neither provide information on clinical features of the disease (type of diabetes, age of onset, duration of the disease) nor whether risk control targets have been met and the GCI is an indicator only based on screening guidelines, nor on treatment recommendations. However, better quality of the process of care often translates into better performance as regards attainment of treatment goals [24], and the final hard outcomes considered, death and CV incidence, enabled us to look at the result beyond any reasonable doubt. Second, the way we classified the levels of care could have introduced some differential misclassification; persons severely ill for conditions other than diabetes (as persons with end stage chronic diseases) are more likely to be poorly cared according to diabetes GLs and thus included in the ONLY GP model of care. This could explain, at least in part, the high mortality rates in this group, and the less steep difference in incidence; given the administrative nature of our data set we were only able to adjust for diagnosed cardiovascular diseases, but not for other severe clinical conditions, so residual confounding could explain our results. However, the presence of a strong bias should result in a steeper mortality in the first months of the follow up in the ONLY GP group, while survival curve in the ONLY GP group shows a regular slope during the whole 4 years of follow-up; moreover, differential misclassification unlikely affects the other three levels. Self-motivation, and willingness to be treated and followed up, could be possible indication bias leading to the best outcomes of the GP AND SPECIALIST, WITH GCI group. In other words, patients who are more spontaneously prone to adhere to the best management of diabetes could have benefited from this predisposition, regardless of the type of care. In this regards a counterpoint is that the adjustment for education level, which is a known proxy of spontaneously adherence to better care [25], could have limited this interference.

A reasonable conclusion is that these findings suggest that a shared care based on both patient management by diabetes clinic and GP in a joint way, has some advantage in treating diabetes and, probably, chronic illnesses. GPs are not accustomed to active medicine and still perform a sort of “on demand“ medicine with no structured recall [26], a particularly effective tool in chronic illness and diabetes [6]; on the other hand specialists are more at ease with education, with periodical recall and start earlier effective therapies [10], [19]. Shared pathways assessing “who does what” at any time of the course of the disease could be valuable tools to plan effective population-based intervention on diabetes. Finally, as attending diabetes clinics alone without GL-screening results in worse health outcomes, more efforts to promote GLs adherence among GPs and diabetologists alike are needed.
